# First culture-confirmed melioidosis case in Mozambique: a wakeup call for better diagnostics and clinical awareness

**DOI:** 10.1093/omcr/omag069

**Published:** 2026-05-10

**Authors:** Shehezady Charfudine Cruz, Cedalia Raimundo, Leonel Andela, Joaquim Marcos, Nafital Joao, Cecile Joyce, Zaid Amad Seni, Montserrat Barrios, Astan Dicko, Celso Monjane, Agnese Comelli, Fabiola Gordillo, Pilar Garcia-Vello

**Affiliations:** Médecins Sans Frontières, Operational Centre Brussels (OCB), Av. Mártires da Revolução, 4VXH+R28 Beira, Mozambique; Beira Central Hospital – Ministry of Health (MISAU), Avenida Jaime Sigauque, 5R3R+WH Beira, Mozambique; Beira Central Hospital – Ministry of Health (MISAU), Avenida Jaime Sigauque, 5R3R+WH Beira, Mozambique; Beira Central Hospital – Ministry of Health (MISAU), Avenida Jaime Sigauque, 5R3R+WH Beira, Mozambique; Médecins Sans Frontières, Operational Centre Brussels (OCB), Av. Mártires da Revolução, 4VXH+R28 Beira, Mozambique; Médecins Sans Frontières, Operational Centre Brussels (OCB), Av. Mártires da Revolução, 4VXH+R28 Beira, Mozambique; Médecins Sans Frontières, Operational Centre Brussels (OCB), Av. Mártires da Revolução, 4VXH+R28 Beira, Mozambique; Médecins Sans Frontières, Operational Centre Brussels (OCB), Av. Mártires da Revolução, 4VXH+R28 Beira, Mozambique; Médecins Sans Frontières, Middle East Medical Unit (MEMU), Hamra Street, Beirut, Lebanon; Instituto Nacional de Saude, Marracuene District, Maputo, Mozambique; Médecins Sans Frontières, Middle East Medical Unit (MEMU), Hamra Street, Beirut, Lebanon; Médecins Sans Frontières, Middle East Medical Unit (MEMU), Hamra Street, Beirut, Lebanon; Médecins Sans Frontières, Middle East Medical Unit (MEMU), Hamra Street, Beirut, Lebanon; Médecins Sans Frontières, Luxembourg Operational Research and Epidemiology Support Unit (LuxOR), 68 Rue de Gasperich, 1617 Gasperich Luxembourg, Luxembourg

**Keywords:** melioidosis, emerging infection, *Burkholderia pseudomallei*, HIV, sepsis

## Abstract

Melioidosis is a disease caused by *Burkholderia pseudomallei* and is an underrecognised cause of severe infection in sub-Saharan Africa. In Mozambique, where infectious diseases are prevalent, and laboratory capacity remains limited, it has not previously been reported. We report the first culture-confirmed case of melioidosis in a 37-year-old man living with advanced HIV. He presented with fever, diffuse ulcerative skin lesions, and pulmonary infiltrates, and was empirically treated for sepsis. He died on day 15 of hospitalisation before culture results were available. The isolate was identified as *B. pseudomallei* susceptible (increased exposure) to ceftazidime and susceptible to meropenem. The identification of melioidosis represents a sentinel case that suggests melioidosis is likely underdiagnosed in Mozambique. Increasing clinical awareness, strengthening diagnostic capacity, and integrating *B. pseudomallei* into differential diagnoses of refractory sepsis, tuberculosis-like illness, and ulcerative skin disease in immunocompromised patients should be a priority.

## Introduction

Melioidosis is an infectious disease caused by *B. pseudomallei*, a Gram-negative environmental bacterium that can cause severe, often fatal infections, particularly in immunocompromised patients. It is primarily acquired via percutaneous inoculation, inhalation, or ingestion of contaminated soil or water; human-to-human transmission is extremely rare [1]. Its clinical manifestation is non-specific and can easily be confused with tuberculosis, sepsis, or other bacterial infections. This contributes to underdiagnosis and underreporting of this serious condition [1]. Early diagnosis of melioidosis is essential because *B. pseudomallei* is intrinsically resistant to many drugs that are commonly used as empiric treatment of severe infections in sub-Saharan Africa [1].

Global modelling estimates suggest that around 165 000 new human cases and roughly 89 000 deaths occur annually. Melioidosis is considered endemic in Southeast Asia and Northern Australia. Large regions of Africa are considered environmentally suitable for *B. pseudomallei*, but the limited diagnosis capacity and clinical awareness hamper its identification [2–4]. Recent reports, including Médecins Sans Frontières (MSF) first confirmation of melioidosis in Mali [5], highlight that the burden in Africa is underestimated [2–4].

To date, Mozambique has had no documented cases of melioidosis. We report the first blood culture-confirmed case of melioidosis identified at Beira Central Hospital in May 2025. This case provides sentinel evidence that melioidosis may be an unrecognised issue in Mozambique. This report aims to raise clinical awareness, advocate for the integration of *B. pseudomallei* into diagnostic algorithms, and reinforce the importance of enhanced microbiological capacity, surveillance, and antimicrobial stewardship.

## Case report

A 37-year-old HIV-positive male fisherman from rural Sofala Province was admitted to Beira Central Hospital (BCH) in April 2025 with a five-day history of fever, malaise, diffuse pruritic skin lesions, and a genital ulcer. His antiretroviral therapy had been interrupted for more than three months, with no other chronic illnesses or recent travel recorded. On admission, he appeared febrile, acutely ill, and septic, with marked pallor, extensive dermatological involvement and signs of chronic malnutrition.

Physical examination revealed widespread desquamative and ulcerative lesions with purulent discharge on the upper and lower limbs, trunk, and neck. The lesions had different morphologies, including ulcerative plaques and crusted areas. A painful penile ulcer was also observed. Lung auscultation showed diffuse bilateral crackles, consistent with lower respiratory tract involvement. No focal neurologic deficits were noted at the time of examination. Laboratory findings revealed a total leucocytosis of 14.900, severe anaemia (haemoglobin 5.4 g/dl), CD4 count > 200 cells/μl (Visitect), and creatinine 158.27 umol/l (eGFR results 49 ml/min). A chest X-Ray showed signs of pneumonia. Initial differential diagnoses included: severe pneumonia with sepsis, Norwegian scabies, chronic malnutrition, and other advanced HIV related conditions. Empirical 2 g/day of ceftriaxone was started to treat severe pneumonia with sepsis (Table 1).

**Table 1:** Clinical timeline of the first culture-confirmed melioidosis case in Mozambique.

Hospital day	Key events
Day 0	Admission with fever, diffuse ulcerative skin lesions, genital ulcer, and pulmonary crackles. Ceftriaxone initiated for presumed severe pneumonia with sepsis
Day 4	Lack of improvement; blood culture collected. Antibiotics switched to ceftazidime
Days 5–7	Mild transient clinical improvement
Days 8–12	Progressive clinical deterioration despite supportive care
Day 13	Abrupt clinical deterioration
Day 14	Second blood culture collected
Day 15	The patient died before culture results were available
Day 21	Blood culture identified a Gram-negative bacterium
Day 23	Isolate identified as *Burkholderia pseudomallei*

On day 4, due to a lack of improvement, blood culture was requested, and antibiotic treatment was switched to ceftazidime 3 g/day 3 times daily. Mild, temporary clinical improvement was noted with lower fever peaks, improvement in mental state and pulmonary findings.

During days 8-12, there was a gradual deterioration despite the supportive care, including blood transfusion and nutritional supplementation. Microbiology results were not available. It was not possible to prescribe carbapenems due to a stockout. Ceftazidime was continued (Table 1).

On day 13, there was an abrupt deterioration, and on day 14, a second blood culture was collected. On day 15, the patient died before blood culture results were available (Table 1).

Following prolonged incubation, this second blood culture sample yielded a viable isolate. Blood cultures were processed manually, and after seven days of incubation at 33–37°C, the bottles flagged positive for a Gram-negative bacillus (day 21) (Table 1). Subculture onto blood agar and MacConkey agar for 24 hours yielded small to medium-sized, smooth, oxidase-positive colonies that were initially white and non-lactose fermenting, with a greenish discolouration developing with age on chromogenic media. Afterwards, colonies were inoculated via API 20 NE for biochemical identification and confirmed the presence of *B. pseudomallei*. Antimicrobial susceptibility testing (Kirby–Bauer, EUCAST) showed resistance to multiple agents but susceptibility (increased exposure) to ceftazidime and susceptibility to meropenem, typical of *B. pseudomallei*. Identification was verified by the BCH laboratory under MSF supervision and confirmed by the microbiology referent. Identification was performed using validated methods under MSF-supervised quality assurance systems.

## Discussion

This case represents the first documented culture-confirmed melioidosis in Mozambique and provides important sentinel evidence that *B. pseudomallei* may be present in the country. Given the patient’s environmental exposure (fishing in rural flood-prone areas), immunosuppression, and clinical presentation with pulmonary involvement and ulcerative skin lesions, the diagnosis is biologically plausible and consistent with classical melioidosis presentation. Laboratory identification of *B. pseudomallei* can be challenging; however, the risk of misidentification was minimised through morphology, oxidase testing, characteristic resistance patterns, and biochemical profile.

Several factors likely contributed to the fatal outcome, including nonspecific symptoms, limited awareness, poor antimicrobial and diagnostic stewardship, and lack of adherence to antiretroviral therapy (ART). Similar diagnosis constraints have been reported across sub-Saharan Africa, where melioidosis is often misdiagnosed as tuberculosis or bacterial sepsis, reflecting broader systemic challenges [2–5]. Melioidosis should be included in the differential diagnosis of sepsis, tuberculosis, and disseminated skin/soft-tissue lesions in Mozambique and potentially in other tropical areas of sub-Saharan Africa.

The treatment of melioidosis should consist of an initial intravenous ceftazidime or a carbapenem for at least 10–14 days, followed by oral trimethoprim–sulfamethoxazole for 3–6 months [6]. However, gaps in antimicrobial and diagnostic stewardship contributed to the fatal outcome. The empiric switch to ceftazidime was appropriate for severe Gram-negative sepsis, but suboptimal dosing and the inability to escalate to meropenem due to a national stockout reduced therapeutic options. Poor antimicrobial stewardship and antibiotic shortages are global issues that are exacerbated in humanitarian settings [7].

The delays in blood culture collection, together with the late availability of results, highlight the need to invest in pragmatic approaches, such as long-term staff capacity building and innovative solutions [7, 8]. Strengthening laboratory capacity is critical for improving the detection of *B. pseudomallei* and other emerging pathogens.

Although a direct association between HIV and melioidosis has not been established, there are many case reports and case series describing melioidosis in people living with HIV, particularly with advanced immunosuppression or interrupted antiretroviral therapy [9, 10]. This case report contributes to that body of evidence. Poor ART adherence remains a recurrent challenge in Mozambique, influenced by multiple structural, social, and health-system factors. Stigma, long travel distances to health facilities, transportation constraints, and overcrowded services often hinder regular follow-up as well as socioeconomic hardship [11].

Overall, this case demonstrates the importance of including melioidosis in clinical algorithms to strengthen laboratory diagnostic capacity, ensure antimicrobial availability, and improve stewardship and HIV-care continuity, thereby optimising outcomes for severe infections such as melioidosis in Mozambique and the broader region.
